# Long-term quality of life in premenopausal women with node-negative localized breast cancer treated with or without adjuvant chemotherapy

**DOI:** 10.1054/bjoc.2000.1337

**Published:** 2000-08-16

**Authors:** F Joly, M Espié, M Marty, J-F Héron, M Henry-Amar

**Affiliations:** Service de Recherche Clinique, Service d’Oncologie, Centre François Baclesse, Route de Lion-sur-Mer, Caen cedex 5, 14076, France; Service d’Oncologie Médicale, Hôpital Saint Louis, Paris cedex 10, 75475, France

**Keywords:** localized breast cancer, clinical trial, premenopausal node-negative women, adjuvant chemotherapy, long-term quality of life

## Abstract

Our purpose was to evaluate the late physical and psychosocial difficulties of premenpausal patients treated for a localized breast cancer and to weigh the impact of chemotherapy on long-term quality of life. Two self-administered questionnaires, the EORTC core QLQ-C30 and the breast module (BR23) were mailed to 179 premenopausal node-negative women continuously disease-free, previously enrolled in a trial testing the efficacy of adjuvant CMF chemotherapy (Espié et al, 1997). The core questionnaire evaluates the physical, role, emotional, cognitive and social functioning and global health status. The breast module includes four functional scales: body image, sexual functioning, sexual enjoyment and future perspective. It also includes symptom scales such as arm or breast symptoms. Some specific professional and social states were added. 119 (68%) patients (mean age 54 years, range 30–69) participated. Mean follow-up time since diagnosis was 9.6 years (4–16). 68% had conservative and 32% radical surgery (with reconstructive surgery in 50%). CMF was given to 77 (65%) patients. Irradiation was administered in 75% of patients irrespective of adjuvant therapy. QLQ-C30 scale scores were similar in patients who had or had not received chemotherapy. Disturbance in body image, sex life and breast symptoms did not differ between patients who had or had not received adjuvant CMF. No major socioprofessional difficulties were reported except problems in borrowing from banks not related to past chemotherapy. With long follow-up, most premenopausal women treated for a localized breast cancer cope with the disease and its treatments. Adjuvant CMF chemotherapy does not appear to impair quality of life nor social and professional life in these patients. © 2000 Cancer Research Campaign


*British Journal of Cancer* (2000) **83**(5), 577–582
© 2000 Cancer Research Campaign
doi: 10.1054/ bjoc.2000.1337, available online at http://www.idealibrary.com on IDEAL®

### Long-term quality of life in premenopausal women with node-negative localized breast cancer treated with or without adjuvant chemotherapy

**F Joly[1,2], M Espié[3], M Marty[3], J-F Héron[2] and M Henry-Amar[1]**

[1]Service de Recherche Clinique; [2]Service d'Oncologie, Centre François Baclesse, Route de Lion-sur-Mer, 14076 Caen cedex 5, France; [3]Service d'Oncologie Médicale, Hôpital Saint Louis, 75475 Paris cedex 10, France

**Summary** Our purpose was to evaluate the late physical and psychosocial difficulties of premenpausal patients treated for a localized breast cancer and to weigh the impact of chemotherapy on long-term quality of life. Two self-administered questionnaires, the EORTC core QLQ-C30 and the breast module (BR23) were mailed to 179 premenopausal node-negative women continuously disease-free, previously enrolled in a trial testing the efficacy of adjuvant CMF chemotherapy (Espié et al, 1997). The core questionnaire evaluates the physical, role, emotional, cognitive and social functioning and global health status. The breast module includes four functional scales: body image, sexual functioning, sexual enjoyment and future perspective. It also includes symptom scales such as arm or breast symptoms. Some specific professional and social states were added. 119 (68%) patients (mean age 54 years, range 30–69) participated. Mean follow-up time since diagnosis was 9.6 years (4–16). 68% had conservative and 32% radical surgery (with reconstructive surgery in 50%). CMF was given to 77 (65%) patients. Irradiation was administered in 75% of patients irrespective of adjuvant therapy. QLQ-C30 scale scores were similar in patients who had or had not received chemotherapy. Disturbance in body image, sex life and breast symptoms did not differ between patients who had or had not received adjuvant CMF. No major socioprofessional difficulties were reported except problems in borrowing from banks not related to past chemotherapy. With long follow-up, most premenopausal women treated for a localized breast cancer cope with the disease and its treatments. Adjuvant CMF chemotherapy does not appear to impair quality of life nor social and professional life in these patients. © 2000 Cancer Research Campaign

**Keywords**: localized breast cancer; clinical trial; premenopausal node-negative women; adjuvant chemotherapy; long-term quality of life

In localized breast cancer treatment is given with a curative intent. While much attention has focused on acute treatment-related toxicity and its impact on patients' quality of life during active cancer treatment, there is less knowledge on the rehabilitation and quality of life outcomes in women with no evidence of disease many years after treatment. In breast cancer survivors, the evaluation of the impact of long-term treatment side-effects on quality of life is important in identifying difficulties, informing patients and minimizing and preventing problems (Dow et al, 1996; Hietanen, 1996; Bernhard et al, 1997). Many studies have been conducted on quality of life and psychological distress after mastectomy or lumpectomy. Their results are all the more contradictory because in most studies, the patient follow-up was limited (less than 2 years) (Knobf, 1986; Kiebert et al, 1991; Maunsell et al, 1993; van Dam et al, 1998). Much less attention has been paid to the impact of adjuvant therapy (Maguire et al, 1980; Berglund et al, 1991; Schover et al, 1995; Hürny et al, 1996; Ganz et al, 1998*a*). Nowadays, more and more patients are given adjuvant chemotherapy (Hebert-Croteau et al, 1999). While impact of adjuvant chemotherapy on overall survival of women with node-negative localized breast cancer is still limited, improvement of disease-free survival has led to a consensus favouring such adjuvant chemotherapy (Goldhirsch et al, 1995; Early Breast Cancer Trialist's Collaborative Group, 1998). Thus, the evaluation of changes possibly induced by chemotherapy on the family, social and professional life of these premenopausal women could affect the consensus. A cross-sectional study was therefore conducted with two objectives: first, to evaluate the impact of breast cancer and its treatment on quality of life and rehabilitation in long-term survivors and secondly, to explore late sequelae induced by adjuvant chemotherapy in patients enrolled in a clinical trial.

*Received 6 May 1999*
*Revised 18 April 2000*
*Accepted 12 May 2000*

*Correspondence to:* F Joly

### PATIENTS AND METHODS

### Selection of patients

The study was conducted among long-term survivors who were initially enrolled in a prospective controlled clinical trial conducted from 1980–1991 at the Polyclinique d'Oncologie Médicale, Hôpital Saint-Louis, Paris, France. The trial compared adjuvant CMF (cyclo-phosphamide, methotrexate, fluorouracil) chemotherapy, given for 6-monthly courses, to no adjuvant chemotherapy (Espié et al, 1997). Premenopausal patients with localized node-negative breast adenocarcinoma were eligible for the trial, in which 335 women were randomized. Patients were eligible for the present study if they fulfilled the following criteria: treated and followed at the Hôpital Saint-Louis, free of disease since the end of initial therapy, and without history of second malignancy. Of the 335 randomized patients, 24 were not treated at Hôpital Saint-Louis, 99 had relapsed, 32 were lost to follow-up, and one had developed a second malignancy. Therefore, 179 patients remained eligible for the study. Among these 179 patients 111 (62%) were given adjuvant CMF, a figure which parallels the better treatment failure-free survival (TFFS) rate observed in the chemotherapy arm compared with the rate in the no adjuvant chemotherapy arm (10-year TFFS: 65% vs 52%, $P = 0.03$).

### Procedure

From November 1996 to February 1997, a written invitation to participate in the study was mailed to the 179 eligible patients, and was followed, 2–3 weeks later, by a letter including two self-administered questionnaires. A reminder (by mail) was sent after 1 month where necessary, and reasons for non-participation were collected. The questionnaires were used to assess the patients' quality of life and the difficulties they may have encountered since their disease and its treatment. The first questionnaire was the French language validated translation of the European Organization for Research and Treatment of Cancer (EORTC) QLQ-C30 core questionnaire (Aaronson et al, 1993) with the breast module (EORTC QLQ-BR23) (Sprangers et al, 1996) after copyright was obtained. The EORTC QLQ-C30 core questionnaire explores the following functional areas: physical, role, emotional, cognitive and social functioning, as well as global health status. It also includes a number of multi-item scales and single items which assess a range of physical symptoms (fatigue, nausea and vomiting, pain, dyspnoea, sleep disturbance, loss of appetite, constipation, diarrhoea), as well as financial difficulties. The breast-specific module includes four functional scales (body image, sexual functioning, sexual enjoyment and future perspective) and a number of symptom scales and items (arm and breast symptoms, systemic therapy side-effects, upset caused by hair-loss). For functional scales, scores computed range from 0–100, with the higher scores representing a higher level of functioning. For item scales relative to symptoms and financial impact, scores computed range from 0–100, with higher scores representing a higher level of symptomatology or problems. The reliability of the core questionnaire (i.e. internal consistency measured by Cronbach's alpha coefficient) ranged from 0.53 for role functioning to 0.94 for global health status (Osoba et al, 1994). Although the questionnaire was designed to address patient status during the week before interview, it also advised that some items could be irrelevant. At the time of the study, no questionnaire was available in French to evaluate the social and professional status of long-term cancer survivors, as well as their medica 1 consumption. Therefore, a second questionnaire (Life questionnaire), used previously in a survey on long-term survivors of Hodgkin's disease (Joly et al, 1996), was specifically compiled and tested on 20 patients prior to the study. Most items addressed in this questionnaire were objective, concerned education grade, marital status, number of children, leisure occupations, life insurance problems, employment and medica 1 consumption and family and social relationships. In addition, items concerning memory of the chemotherapy experience were added, and patients were asked to provide their opinions on the study. All items were addressed for the status at the time of breast cancer diagnosis and for the status in 1997. These two self-administered questionnaires were usually completed within 45 min. Clinical data was issued from medical charts. It concerned the date of diagnosis, the dates of beginning and end of treatment, clinical stage, type of initial surgery (lumpectomy, mastectomy with or without breast reconstruction), type of radiation therapy, type of chemotherapy, and clinical outcome including date of last visit.

### Statistical analysis

Tests used for comparison of means included the Student test and the Mann–Whitney test. Those used for proportion comparisons were the $\chi^2$-test and the Fisher exact test. Multivariate analysis of variance and covariance (ANCOVA) was used for group-wise comparisons of the quality of life-related scores with Bonferroni correction. In the analysis, adjustments were made on comorbid medical illnesses, age and education grade as appropriate. No adjustment was made on treatment type (surgery, irradiation) since it was similarly distributed among CMF and no adjuvant chemotherapy groups. Two-sided $P$-values were used as descriptive statistics to identify associations in the observed results. Statistical tests were considered significant for $P < 0.01$. Missing data was infrequent (< 10%) except for questions related to sexuality, in particular sexual enjoyment where 25% of data was missing. When missing, data was replaced by the mean value of the corresponding item among respondents. Programs 4F, 3D, 7D and 2V of the BMDP statistical software were used to analyse the data (Dixon, 1992).

### RESULTS

### Participation

Among the 179 eligible patients, 122 (68%) agreed to participate, 98 spontaneously and 24 after a reminder. Of the 57 non-participants, two refused, 19 were lost to follow-up, and 36 did not reply (Figure 1). Demographic, clinical (family history of breast cancer, tumour size), treatment characteristics (type of surgery, proportion of patients given CMF) and follow-up time did not statistically differ between participants and non-participants. Of the 122 participants, three had relapsed within a year before the questionnaires were sent, and were excluded from the study leaving 119 patients eligible.

### Patient characteristics and treatment

Clinical characteristics are listed in Table 1. All patients had surgery with axillary clearance. Nineteen patients had breast reconstruction 0–6 years after mastectomy (mean 1.3 years) and 15 used external breast prosthesis. Irradiation was given to 75% of patients in the CMF group and in 67% of patients in the no adjuvant chemotherapy group. Lumpectomy was performed in 69% and 64% of patients in the two arms, respectively. For patients who had mastectomy, breast reconstruction was carried out in 50% of patients in the two arms. As per protocol, no patients received adjuvant hormonotherapy. After the diagnosis of breast cancer and its treatment, 48% of women (45% and 54% in the CMF and the no adjuvant chemotherapy group, $P = 0.50$) had cancer-unrelated medical history including rheumatoid disease in 30%, cardiovascular disease in 12%, metabolic disorders in 12% and digestive disease in 6%. Twenty six percent of patients in the CMF group and 28% of those in the no adjuvant chemotherapy group had a family history of breast cancer.

 © 2000 Cancer Research Campaign

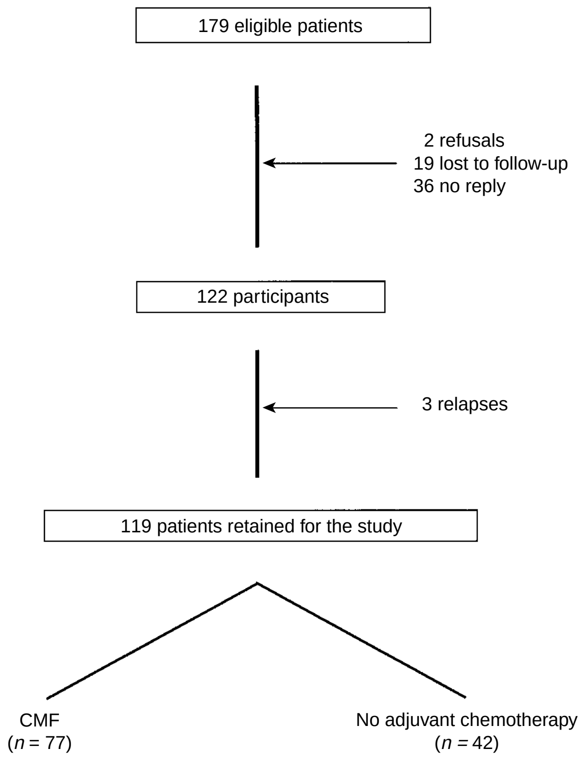

**Figure 1** Participation in study

The mean follow-up time from initial treatment was 9.6 years (range 4–16). Forty six percent of patients were qualified (36% were of management grade or higher). Most patients were married and 84% had children, including three children born after treatment was completed. At time of interview, 83% of patients were post-menopausal. CMF patients were younger than those who were not given adjuvant chemotherapy (53 years vs 55 years, $P$ = 0.04). Follow-up time was shorter in the CMF group (9.1 years vs 10.5 years, $P$ = 0.01) (Table 2).

### Long-term quality of life

Seventy nine percent of patients reported a good appraisal of the study while four (3%) patients expressed painful memories induced by the questionnaires.

**Table 1** Clinical characteristics ($n$ = 119)

| Variable | *n* | % |
|---|---|---|
| Family history of breast cancer | 32 | 27 |
| Clinical stage (UICC) | | |
| T0 | 11 | 8 |
| T1–T2 | 103 | 87 |
| T3–T4 | 5 | 5 |
| Type of surgery | | |
| Lumpectomy | 81 | 68 |
| Mastectomy | 19 | 16 |
| Mastectomy with breast reconstruction | 19 | 16 |
| External beam irradiation[a] | 86 | 72 |
| Brachytherapy (boost)[b] | 75 | 63 |
| Adjuvant CMF[c] chemotherapy | 77 | 65 |
| Mean treatment duration, in days (range) | 59 (36–87) | – |
| Menopausal status at interview | 99 | 83 |

[a]45 Gy, standard dose; [b]20 Gy, standard dose; [c]cyclo-phosphamide 400 mg $m^{-2}$, methothrexate 40 mg $m^{-2}$, fluorouracil 400 mg $m^{-2}$, day 1 and day 8, q 28 days for six courses

### *Quality of life assessed by the EORTC questionnaires*

All generic quality of life scale scores were similar in patients who were or were not given chemotherapy (Table 3). Specific dimensions and symptoms of the breast module (body image and sexuality) were not influenced by chemotherapy (Table 4). Education grade correlated with score of future perspective ($r$ = 0.2, $P$ = 0.1) and those of arm and breast symptoms: the higher the education grade, the higher the functional scale score (future perspective) and the lower the symptom scale scores (arm and breast symptoms). Follow-up time since initial treatment (less or more than 10 years) only influenced one quality of life scale score. In CMF patients, the social functioning scale score was higher in patients with follow-up of more than 10 years (97 vs 85, $P$ = 0.006)

### *Life questionnaire data*

At time of study, 44% of patients reported treatment-related sequelae of any type or intensity (40% in the CMF group and 51% in the no adjuvant chemotherapy group, $P$ = 0.53). Chronic fatigue was reported by 19% of CMF patients and by 31% of patients with no adjuvant chemotherapy ($P$ = 0.12). Overall, 56% of patients gained weight after treatment (57% in the CMF and 55% in the no adjuvant chemotherapy groups). Weight-gain was considered chemotherapy-related by 2% of patients, related to chemotherapy-induced menopause by 23% of patients, and was a consequence of change in lifestyle in 31% of patients. Dysfunction in sexual life was reported by 30% (16 CMF and 20 no adjuvant chemotherapy)

**Table 2** Demographic characteristics at interview ($n$ = 119)

| | No chemotherapy (*n* = 42) | CMF *n* = 77) | *P* value |
|---|---|---|---|
| Mean age in years (range) | 55 (40–69) | 53 (30–66) | 0.04 |
| Mean time from treatment in years (range) | 10.5 (6–16) | 9.1 (4–16) | 0.01 |
| Education grade (%) | | | |
| High | 21 (50) | 33 (43) | |
| Middle | 18 (43) | 35 (45) | |
| Low | 3 (7) | 9 (12) | 0.60 |
| Annual family income, in Euro[a] > 18 462[b] (%) | 26 (61) | 56 (73) | 0.30 |
| Currently married (%) | 35 (83) | 62 (81) | 0.90 |
| Children (%) | 34 (81) | 66 (86) | 0.70 |
| Mean number (range) | 1.5 (1–4) | 1.6 (1–5) | – |

[a]Data available in 109 patients; [b]One Euro = 6.57957 French francs

*© 2000 Cancer Research Campaign* 

**Table 3** Chemotherapy impact on quality of life (EORTC QLQ-C30 core questionnaire): mean values (standard deviation)[a]

| | No chemotherapy (*n* = 42) | CMF (*n* = 77) | *P* value[b] |
|---|---|---|---|
| Functional scales[c] | | | |
| Physical functioning | 80 (20) | 84 (15) | 0.30 |
| Role functioning | 63 (51) | 62 (62) | 0.80 |
| Emotional functioning | 68 (29) | 74 (24) | 0.40 |
| Cognitive functioning | 83 (47) | 68 (20) | 0.04 |
| Social functioning | 86 (26) | 89 (21) | 0.66 |
| Global health status | 70 (18) | 75 (18) | 0.20 |
| Symptom scales/items[d] | | | |
| Fatigue | 28 (24) | 20 (21) | 0.12 |
| Nausea and vomiting | 15 (45) | 3 (10) | 0.04 |
| Pain | 21 (31) | 16 (21) | 0.50 |
| Dyspnoea | 22 (24) | 16 (23) | 0.20 |
| Sleep disturbance | 38 (39) | 24 (31) | 0.05 |
| Appetite loss | 12 (24) | 4 (16) | 0.09 |
| Constipation | 19 (32) | 15 (28) | 0.68 |
| Diarrhoea | 4 (12) | 4 (11) | 0.90 |
| Financial difficulties | 5 (23) | 7 (19) | 0.55 |

[a]With minor variations in sample size because of missing data; [b]Covariance analysis (ANCOVA) with correction for co-morbid medical illness, menopausal status at interview and time since treatment; [c]A high scale score represents a high level of functioning; [d]A high scale score represents a high level of symptomatology or problem

**Table 4** Chemotherapy impact on quality of life (EORTC breast module): mean values (standard deviation)[a]

| | No chemotherapy (*n* = 42) | CMF (*n* = 77) | *P* value[b] |
|---|---|---|---|
| Functional scales[c] | | | |
| Future perspective | 57 (34) | 55 (36) | 0.60 |
| Body image | 80 (26) | 76 (29) | 0.70 |
| Sexual functioning | 26 (28) | 35 (26) | 0.40 |
| Sexual enjoyment | 47 (21) | 60 (32) | 0.10 |
| Symptom scales/items[d] | | | |
| Breast symptoms | 18 (21) | 20 (21) | 0.60 |
| Arm symptoms | 19 (20) | 18 (20) | 0.80 |

[a]With minor variations in sample size because of missing data; [b]Covariance analysis (ANCOVA) with correction for age and co-morbid medical illness, menopausal status at interview and time since treatment; [c]A high scale score represents a high level of functioning; [d]A high scale score represents a high level of symptomatology or problem of patients. Among the 16 CMF patients, sexual dysfunction was considered as a consequence of chemotherapy-induced menopause in four. No difference in the proportion of patients with modifications in relationships with partner (45% vs 52%), children (21% vs 22%) or friends (24% vs 36%) was observed in the CMF group and the no adjuvant chemotherapy group, respectively. Breast cancer was responsible for separation or divorce in three women only (including two CMF patients). Sentimental ties with partner were strengthened in 29% and 39% of CMF and no adjuvant chemotherapy patients, respectively ($P = 0.5$).

### *Professional and social difficulties issued from the life questionnaire*

At diagnosis, 78% ($n = 60$) of CMF patients and 64% ($n = 27$) of no adjuvant chemotherapy patients were professionally active while, at time of interview, 62% ($n = 48$) and 40% ($n = 17$) continued to work, respectively. Among the 22 patients who stopped working, 19 had retired (11 and eight patients, respectively), and three were unable to work while in complete remission (two and one patients, respectively). Between breast cancer therapy and 1997, 28% ($n = 25$) of women at work had changed their employer or the position occupied. Personal choice was the main reason while two women were dismissed because of their disease (one in each group). In four patients, the work-post was adapted because of surgery or irradiation-related arm sequelae. Changes in professional projects were independent of previous chemotherapy, age and education grade. Less professional ambition was expressed by 66% and 67% of CMF and no adjuvant chemotherapy patients, respectively, while the disease had reinforced the professional ambitions of the remaining patients at work. Fourteen (12%) patients (nine CMF and five no adjuvant chemotherapy) reported financial difficulties because of breast cancer; in six of them (three and three) the problem was still present in 1997. Companies refused life insurance to eight of 65 women requesting it. Both financial and life-insurance difficulties were unrelated to previous chemotherapy or time since therapy. Among the 40 patients who had borrowed

© 2000 Cancer Research Campaign money from banks, 20 (50%) reported difficulties in subscription (higher interest rate, restriction of the contract, refusal) independently of previous chemotherapy.

### DISCUSSION

Many studies have explored quality of life and difficulties experienced by women with breast cancer both during treatment and in the short post-treatment period (Halttunen et al, 1992; Dorval et al, 1998; Ganz et al, 1998*a*). In contrast, no studies have explored long-term consequences of chemotherapy for breast cancer. A study, therefore, was conducted in long-term breast cancer survivors of localized disease, who were enrolled in a clinical trial comparing adjuvant CMF to no adjuvant chemotherapy (Espié et al, 1997). The study focused on node-negative young women with an average follow-up of 10 years. In contrast to published studies in which all non-metastatic breast cancer patients were considered, the study concerns a subgroup of patients for whom the treatment strategy today includes adjuvant chemotherapy (Goldhirsch et al, 1995; Early Breast Cancer Trialist's Collaborative Group, 1998). The results indicate that women have coped with the disease, achieving a satisfactory global quality of life irrespective of the adjuvant treatment administered. Although women report persisting symptoms related to their cancer and its treatment, many positive benefits are gained which help to balance the negative aspects (Dow et al, 1996; Ganz et al, 1998*b*).

Adjuvant chemotherapy has been reported to partially influence quality of life during or after the treatment period (McArdle, 1979; Maguire et al, 1980; Palmer et al, 1980; Hughson et al, 1986; Knobf, 1986; Schover et al, 1995). Its effect is transient and minor compared with patients' adjustment or coping after diagnosis and surgery (Hürny et al, 1996). The limited impact of chemotherapy can relate to the mild physical distress that persists after the treatment, with minimal alteration in life style and little change in sexual relationships (Knobf, 1986). Two–5 years after adjuvant therapy self-rated quality of life in breast cancer patients is generally good, although selected aspects (sexual acceptance by the partner, negative feelings toward sexual relations) appear to be compromised in less than half of the women (Ganz et al, 1998*a*). In the present study, patients who received adjuvant chemotherapy do not report more sexual and body-image dysfunction than those who had not, a result that can be explained by a longer follow-up, with improved coping with time. Another difference concerns tools used to evaluate the sexuality that are very limited in French and probably not very relevant. However, cultural differences in the USA and in Europe cannot be neglected. While body-image problems and sexual disorders are frequently reported (Schover et al, 1995; Dow et al, 1996; Ganz et al, 1996; Barni and Mondin, 1997), the influence of chemotherapy upon sexual satisfaction is not clear (Ganz et al, 1998*b*). Although some physical symptoms (such as prematurely chemotherapy-induced menopause) have the potential to detract from sexual activity, other independent physical and psychological factors may in fact predominate as determinants of sexual functioning, such as age-related changes in desire, arousal and quality of partnered relationships (Ganz et al, 1999).

Nowadays, anthracycline-containing regimens are more commonly used in node-positive premenopausal breast cancer patients, inducing more acute toxicity, such as alopecia and nausea, than CMF. One cannot exclude, in the future, a significant impact of adjuvant chemotherapy on long-term psychological disorders, since more cognitive impairment in patients with anthracycline-containing chemotherapy have been reported (van Dam et al, 1998). Moreover, the difference is higher in patients receiving high-dose chemotherapy than in patients receiving standard-dose, and higher in the latter than in patients without adjuvant chemotherapy.

Arm and breast symptoms may persist many years after the end of treatment. Main complaints concerning pain, weakness, stiffness and some limitation in range of movement are surgery and/or irradiation-related, and not chemotherapy-related (Kiebert et al, 1991; Maunsell et al, 1993). Even if these symptoms are reported often, they seem to only moderately influence the long-term patient quality of life (Craig et al, 1974; Ganz et al, 1996; Dorval et al, 1998; Lindley et al, 1998).

Even 10 years after diagnosis and treatment, other mild social problems or difficulties are still reported. A major difficulty encountered by survivors concerns life insurance (which is mandatory for borrowing money from banks), while difficulties in patient's work are rarely reported (Dow et al, 1996; Ganz et al, 1996). However, the problem appears to be less important in female breast cancer survivors than in men surviving cancer, because a majority of women are married and their spouse can on some occasions request insurance in their own name (Joly et al, 1996). Past adjuvant chemotherapy does not appear to influence problems and refusal from banks and insurance companies, which are more likely to be related to personal history of cancer than to its treatment.

Our study highlights that with long follow-up, most premenopausal node-negative women treated for localized breast cancer cope with the disease and its treatment. In particular, adjuvant CMF chemotherapy does not appear to impair quality of life nor social and professional life in these patients.

### ACKNOWLEDGEMENTS

This study was supported by a grant from the Fondation de France.

### REFERENCES

Aaronson NK, Ahmedzai S, Bergman B, Bullinger M, Cull A, Duez NJ, Filiberti A, Flechtner H, Fleishman SB, de Haes JCJM, Kaasa S, Klee M, Osoba D, Razavi D, Rofe PB, Schraub S, Sneeuw K, Sullivan M and Takeda F (1993) The EORTC QLQ-C30: A quality of life instrument for use in international clinical trials in oncology. *J Natl Cancer Inst* **85**: 365–376

Barni S and Mondin R (1997) Sexual dysfunction in treated breast cancer patients. *Ann Oncol* **8**: 149–153

Berglund G, Bolund C, Fornander T, Rutqvist LE and Sjoden PO (1991) Late effects of adjuvant chemotherapy and postoperative radiotherapy on quality of life among breast cancer patients. *Eur J Cancer* **27**: 1075–1081

Bernhard J, Hürny C, Coates AS, Peterson HF, Castiglione-Gertsch M, Gelber RD, Goldhirsch A, Senn HJ and Rudenstam CM (1997) Quality of life assessment in patients receiving adjuvant therapy for breast cancer: The IBCSG approach. *Ann Oncol* **8**: 825–835

Craig TJ, Comstock GW and Geiser PB (1974) The quality of survival in breast cancer: A case-control comparison. *Cancer* **33**: 1451–1457

Dixon WJ (1992) *BMDP statistical software Release 7.0*. University of California Press: Berkeley

Dorval M, Maunsell E, Deschênes L, Brisson J and Mâsse B (1998) Long-term quality of life after breast cancer: Comparison of 8-year survivors with population controls. *J Clin Oncol* **16**: 487–494

Dow KH, Ferrell BR, Leigh S, Ly J and Gulasekaram P (1996) An evaluation of the quality of life among long-term survivors of breast cancer. *Breast Cancer Res Treat* **39**: 261–273

Early Breast Cancer Trialist's Collaborative Group (EBCTCG) (1998) Polychemotherapy for early breast cancer: An overview of the randomised trials. *Lancet* **352**: 930–942

© 2000 Cancer Research Campaign 

Espié M, Mignot L, Leleu F, De Roquancourt A, Maylin C, Morvan F, Clot P and Marty M (1997) Improved outcome of node negative breast cancer in premenopausal women with adjuvant CMF: Results of a randomized study. *Proc ASCO* **16**: 141 (Abstr)

Ganz PA, Coscarelli A, Fred C, Kahn B, Polinsky ML and Petersen L (1996) Breast cancer survivors: Psychosocial concerns and quality of life. *Breast Cancer Res Treat* **38**: 183–199

Ganz PA, Rowland JH, Meyerowitz BE and Desmond K (1998*a*) Impact of different adjuvant therapy strategies on quality of life in breast cancer survivors. *Recent Results Cancer Res* **152**: 396–411

Ganz PA, Rowland JH, Desmond K, Meyerowitz BE and Wyatt GE (1998*b*) Life after breast cancer: Understanding women's health-related quality of life and sexual functioning. *J Clin Oncol* **16**: 501–514

Ganz PA, Desmond KA, Belin TR, Meyerowitz BE and Rowland JH (1999) Predictors of sexual health in women after a breast cancer diagnosis. *J Clin Oncol* **17**: 2371–2380

Goldhirsch A, Wood WC, Senn HJ, Glick JH and Gelber RD (1995) Fifth international conference on adjuvant therapy of breast cancer, St Gallen, March 1995. International consensus panel on the treatment of primary breast cancer. *Eur J Cancer* **31A**: 1754–1759

Halttunen A, Hietanen P, Jallinoja P and Lonnqvist J (1992) Getting free of breast cancer. An eight-year perspective of the relapse-free patients. *Acta Oncol* **31**: 307–310

Hebert-Croteau N, Brisson J, Latreille J, Gariepy G, Blanchette C and Deschênes L (1999) Time trends in systemic adjuvant treatment for node negative breast cancer. *J Clin Oncol* **17**: 1458–1464

Hietanen PS (1996) Measurement and practical aspects of quality of life in breast cancer. *Acta Oncol* **35**: 39–42

Hughson AV, Cooper AF, McArdle CS and Smith DC (1986) Psychological impact of adjuvant chemotherapy in the first two years after mastectomy. *BMJ* **293**: 1268–1271

Hürny C, Bernhard J, Coates AS, Castiglione-Gertsch M, Peteron HF, Gelber RD, Forbes JF, Rudenstam CM, Simoncini E, Crivellai D, Goldhirsch A and Senn HJ (1996) Impact of adjuvant therapy on quality of life in women with node-positive operable breast cancer. *Lancet* **347**: 1279–1284

Joly F, Henry-Amar M, Arveux P, Reman O, Tanguy A, Pény AM, Lebailly P, Macé-Lesec'h J, Vié B, Génot JY, Busson A, Troussard X and Leporrier M (1996) Late psychosocial sequelae in Hodgkin's disease survivors: A French population-based case-control study. *J Clin Oncol* **14**: 2444–2453

Kiebert GM, de Haes JCJM and van de Velde CJH (1991) The impact of breast-conserving treatment and mastectomy on the quality of life of early-stage breast cancer patients: A review. *J Clin Oncol* **9**: 1059–1070

Knobf MT (1986) Physical and psychologic distress associated with adjuvant chemotherapy in women with breast cancer. *J Clin Oncol* **4**: 678–684

Lindley C, Vasa S, Sawyer WT and Winer EP (1998) Quality of life and preferences for treatment following systemic adjuvant therapy for early-stage breast cancer. *J Clin Oncol* **16**: 1380–1387

Maguire GP, Tait A, Brooke M, Thomas C, Howat JM, Sellwood RA and Bush H (1980) Psychiatric morbidity and physical toxicity associated with adjuvant chemotherapy after mastectomy. *BMJ* **281**: 1179–1180

Maunsell E, Brisson J and Deschenes L (1993) Arm problems and psychological distress after surgery for breast cancer. *Can J Surg* **36**: 315–320

McArdle CS (1979) The emotional and social implication of adjuvant chemotherapy in breast cancer. In *Adjuvant therapy of cancer*, Salmon SE (ed) pp 319–325. Crune and Stratton: Orlando

Osoba D, Zee B, Warr D, Kaizer L and Latreille J (1994) Psychometric properties and responsiveness of the EORTC quality of life questionnaire (QLQ-C30) in patients with breast ovarian and lung cancer. *Qual Life Res* **3**: 353–364

Palmer BV, Walsh GA, McKinna JA and Greening WP (1980) Adjuvant chemotherapy for breast cancer: Side effects and quality of life. *BMJ* **281**: 1594–1597

Schover LR, Yetman RJ, Tuason LJ, Meisler E, Esselstyn CB, Hermann RE, Grundfest-Broniatowski S and Dowden RV (1995) Partial mastectomy and breast reconstruction. A comparison of their effects on psychosocial adjustment, body image, and sexuality. *Cancer* **75**: 54–64

Sprangers MA, Groenvold M, Arraras JI, Franklin J, te Velde A, Muller M, Franzini L, Williams A, de Haes HC, Hopwood P, Cull A and Aaronson NK (1996) The European Organization for Research and Treatment of Cancer breast cancer-specific quality-of-life questionnaire module: First results from a three-country field study. *J Clin Oncol* **14**: 2756–2768

van Dam FS, Schagen SB, Muller MJ, Boogerd W, Wall VD, Droogleever FME and Rodenhuis S (1998) Impairment of cognitive function in women receiving adjuvant treatment for high-risk breast cancer: High-dose versus standard-dose chemotherapy. *J Natl Cancer Inst* **90**: 210–218

© 2000 Cancer Research Campaign
